# Disparity of Gut Microbiota Composition Among Elite Athletes and Young Adults With Different Physical Activity Independent of Dietary Status: A Matching Study

**DOI:** 10.3389/fnut.2022.843076

**Published:** 2022-03-18

**Authors:** Yongjin Xu, Fei Zhong, Xiaoqian Zheng, Hsin-Yi Lai, Chunchun Wu, Cong Huang

**Affiliations:** ^1^Department of Sports and Exercise Science, Zhejiang University, Hangzhou, China; ^2^Department of Neurology and Research Center of Neurology in Second Affiliated Hospital, Key Laboratory of Medical Neurobiology of Zhejiang Province, Interdisciplinary Institute of Neuroscience and Technology, Zhejiang University School of Medicine, Hangzhou, China; ^3^Key Laboratory for Biomedical Engineering of Ministry of Education, College of Biomedical Engineering and Instrument Science, Zhejiang University School of Medicine, Hangzhou, China; ^4^Department of Neurology of Sir Run Run Shaw Hospital, Zhejiang University School of Medicine, Hangzhou, China; ^5^Department of Medicine and Science in Sports and Exercise, Tohoku University Graduate School of Medicine, Sendai, Japan

**Keywords:** gut microbiota, dietary status, physical activity, elite athlete, inflammation, matching study

## Abstract

**Objective:**

This study aimed to investigate the disparity of gut microbiota among elite athletes and young adults with different physical activity independent of dietary status.

**Methods:**

In Hangzhou, China, an age and sex matching study was conducted between April and May 2021. A total of 66 Chinese young adults were recruited in this study and divided into an elite athlete group, physically active group, and physically inactive group. Fecal samples were collected to assess gut microbiota composition. Dietary status was measured using a food-frequency questionnaire. Comparisons in gut microbiota and blood biomarkers among three groups were analyzed by using the analysis of covariance.

**Results:**

The findings depicted a tendency to form clusters for beta diversity among three groups, while no significant difference was observed in both alpha and beta diversity. In the multiple analysis model, by adjusting dietary status, a significantly higher abundance of *Clostridiaceae* (*p* = 0.029) and *Megamonas_rupellensis* (*p* = 0.087) was observed in elite athletes compared to that in general young adults. Furthermore, inflammation-related bacteria such as *Bilophila* (*p* = 0.011) and *Faecalicoccus* (*p* = 0.050) were enriched in physically inactive young adults compared to two other groups. Pearson's correlation analysis showed a positive association between *Bilophila* and circulating white body cell count (*r* = 0.332, *p* = 0.006) and its subtypes including neutrophils (*r* = 0.273, *p* = 0.027), and lymphocytes (*r* = 0.327, *p* = 0.007). *Megamonas_rupellensis* has been shown associated positively with serum lymphocytes levels (*r* = 0.268, *p* = 0.03). Although no significant differences were observed, the elite athletes tended to have lower levels of blood biomarkers of immunity within a normal range, which may reflect a better immune function.

**Conclusion:**

This matching study indicated that physically inactive young adults are more likely to have a lower immune function and a higher abundance of pro-inflammatory gut bacteria than elite athletes and physically active young adults. Dietary status should be considered as an important factor that may affect the association of physical activity with immune function and gut microbiota.

## Introduction

Humans are superorganisms composed of human bodies and commensal microbiota (1). The human gut microbiota is approximately 100 trillion organisms, outnumbering the human cells by an estimated 10-fold (2–4). Increasing evidence suggests that there is a close relationship between gut microbiota composition, inflammation (5, 6), and immunity (7, 8). A systematic review showed that gut microbiota is thought to contribute to subacute systemic inflammatory and it may also be influenced by outcome, thereby reinforcing the disease symptoms (9). Some gut microbiota may be associated with pro-inflammatory effects, while others have anti-inflammatory properties. As potential anti-inflammatory microbiota, *Lachnospiraceae* and *Ruminococcus* are thought to prevent or ameliorate chronic hepatitis B (10). Conversely, pro-inflammatory gut microbiota, such as *Bacteroides fragilis*, were highly enriched in the gut of inflammatory arthritis (11). In addition, the *Bacteroides fragilis* toxin was also reported to be pro-inflammatory and carcinogenic (12). Thus, gut microbiota could be considered an important indicator of infectious and metabolic diseases.

It is well-known that people with high physical activity levels tend to have better immune function and lower levels of inflammation, which may help them improve health status and prevent diseases. Evidence showed that physically active adolescent girls had lower circulating IL-6 concentration compared to their sedentary counterparts (13). Moreover, a recent study reported that higher physical activity levels may be associated with lower salivary C-reactive protein in young adults (14). Studies regarding the relationship of exercise habits with inflammation-related gut microbiota are emerging. The new literature enables a better understanding of the underlying mechanisms of the association between physical activity and inflammation. Higher proportions of *Akkermansiaceae* family and *Akkermansia* genus in elite rugby athletes were detected than in overweight or obese young adults (15). Meanwhile, our previous randomized controlled trial showed that 8 weeks of combined aerobic and resistance exercise not only resulted in improved physical function but also increased abundance of anti-inflammatory bacteria and decreased pro-inflammatory bacteria in physically inactive older women (16). Even with these findings, the evidence exploring the distribution of inflammation-related gut microbiota in people with different physical activity levels is limited, and further studies are warranted.

Conversely, dietary status and nutrient intake are very important and non-negligible indicators when discussing physical activity levels and gut microbiota. Diet is considered to be a pivotal factor affecting the genes and composition of gut microbiota, of which the number, type, and balance of macronutrients (fats, proteins, and carbohydrates) all have a significant impact on gut microbiota (17). In addition, data indicated that the consumption of nuts, oily fish, fruit, vegetables, and cereals is linked to a higher abundance of anti-inflammatory bacteria (18). Meanwhile, dietary status was also shown to be associated with physical activity levels. Previous studies indicated that habitual Mediterranean dietary pattern is associated with more time spent on leisure-time physical activity among older adults (19). Momentary physical activity was concomitant with momentary consumption of both healthy and unhealthy dietary intake in college students (20). Thus, it is hypothesized that dietary status may play a contributory role in the association of physical activity with gut microbiota. However, most previous observational studies and exercise intervention trials have failed to confirm the relationship between physical activity and gut microbiota composition concerning independent of dietary status (15, 16, 21).

Therefore, this matching study aims to investigate the differences in the diversity and composition of gut microbiota among elite athletes and the general young adults with different physical activity levels, as well as explore whether the association between physical activity and gut microbiota are independent of dietary status.

## Materials and Methods

### Participants

An age and sex matching study was conducted between April 2021 and May 2021 in Hangzhou, China, and aimed to examine the difference of gut microbiota, anthropometry, life-behavior habits, and physical performance between athletes and physically active or inactive young adults. All elite athletes are engaged in track and field events (including 400 m, 1,500 m, high jump, long jump, discus, javelin, and pole vault), and had at least 8 years of professional training experience as well as participated in official sports events held by the Chinese Athletics Association or International Association of Athletics Federation.

**Figure 1:**
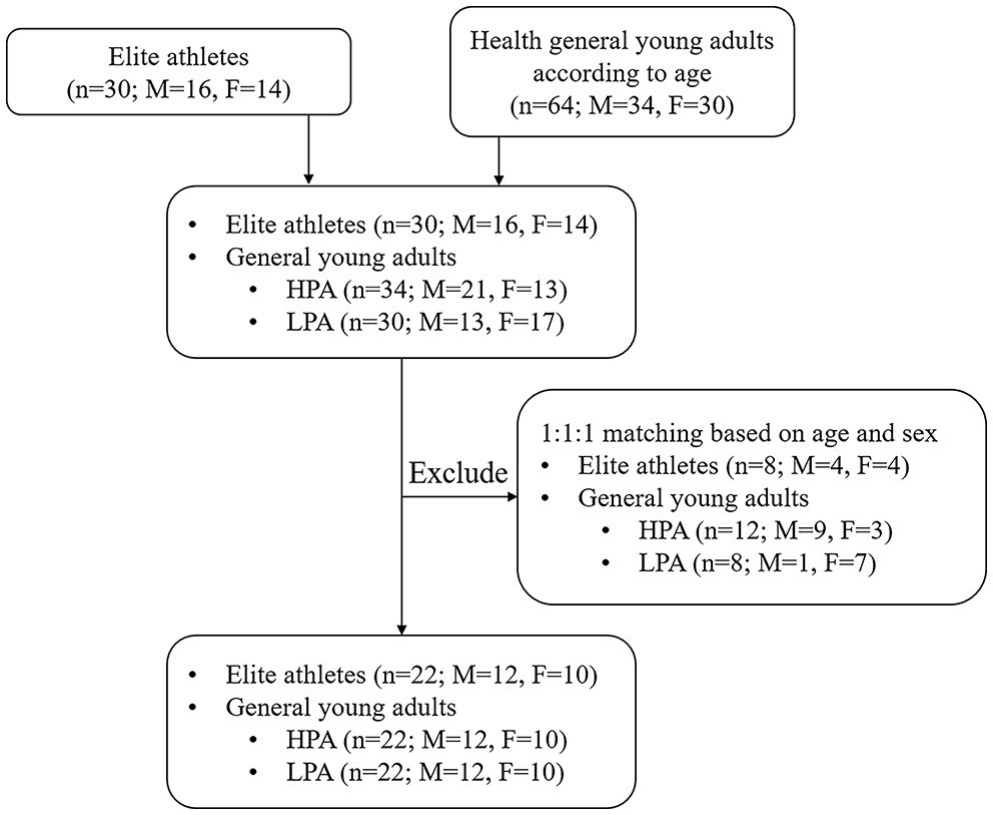
Schematic presentation of the flow of participants through screening.

Thirty young elite athletes were included in this study according to the following inclusion criteria: (1) 18–25 years old; (2) no history of cardiovascular diseases; (3) no history of congenital diseases; (4) no history of physical dysfunction. Participants from the control group included general young adults who met the physical activity recommendation levels according to the guidelines of the American College of Sports Medicine (22). A total of 64 general college students were invited to participate in this study. Among them, 34 students engaged in more than 150 min of moderate-intensity physical activity every week, and 30 students did not engage in any moderate or vigorous-intensity physical activity per day. Physical activity was assessed using the International Physical Activity Questionnaire (IPAQ), which has good reliability and validity for assessing physical activity in healthy adults (23). Thus, 94 elite athletes and college students were allowed to enter the matching process. These participants were then divided into three groups (Athlete group; High physical activity group, HPA; and Low physical activity group, LPA) according to equal proportion principle with age (±24 months) and sex. Finally, a total of 66 young adults were enrolled in the present study (Figure 1). The study protocol and all amendments were approved by the Ethics Committee of the Department of Psychology and Behavioral Sciences, Zhejiang University. All individuals provided written informed consent to participate in the study.

### Dietary Evaluation

In the present study dietary information was collected by food-frequency questionnaire (FFQ25) which was demonstrated to have good reproducibility and validity (24). The FFQ25 primarily investigates the food intake of participants over the past week and contains 25 categories of foods. The frequency of intake of each food is divided into nine levels, from never eating to at least three times a day. The amount of food consumed is divided into five levels, from no more than 50 to at least 250 g a meal. Further, the frequency and amount categories were calculated as times per day by using a midpoint (e.g., “3–4 times per week” was used in the calculations as 3.5 times per week). Finally, all kinds of foods were divided into six groups: cereals, vegetables and fruits, meat, beans and nuts, fatty food, and alcohol.

### Feces Samples Collection and DNA Extraction

The fecal samples were collected by the participants themselves, and the sample collection supplies and methods are uniformly provided and guided by an experimenter before the start of the sample collection. All samples were stored in dry ice at −80°C and transported to Huada Gene Detection Center for DNA extraction. The DNA of the intestinal microbial community was extracted using MagPure Stool DNA KF kit B (Magen, China). The Qubit^®^ dsDNA BR Assay kit (Invitrogen, USA) was used to quantify the DNA with a Qubit Fluorometer. The regions V3–V4 of gut microbiota 16S rRNA genes were amplified using the degenerate PCR primers, 341F (5′-ACTCCTACGGGAGGCAGCAG-3′) and 806R (5′-GGACTACHVGGGTWTCTAAT-3′). Illumina adapter, pad, and linker sequences were added to both forward and reverse primers. A 50 μL reaction containing a 30-ng template, fusion PCR primer, and PCR master mix was used for PCR enrichment. The following were the PCR cycling conditions; 94°C for 3 min; subsequently, 30 cycles of 94°C for 30 s; 56°C for 45 s; 72°C for 45 s, and final extension at 72°C for 10 min. AmpureXP beads were used to purify the PCR products, which were then eluted in the Elution buffer. The Agilent 2,100 bioanalyzer (Agilent, USA) was used to qualify the libraries. Validated libraries were sequenced on the IlluminaMiSeq platform (BGI, Shenzhen, China) according to Illumina's standard procedures, and 2 × 300 bp paired-end reads were generated. The raw reads were filtered to detach adaptors, low-quality, and ambiguous bases to get the tags (25). In addition, The tags were clustered into OTUs using UPARSE software (v7.0.1090) (26). UCHIME (v4.2.40) was used to compare the chimera sequence with the Gold database (27) and classify the representative sequences of OUT by QIIME v1.8.0 (28). All tags were compared with OTU using the USEARCH global (29), and a statistical table of OTU abundance for each sample was obtained. We used MOTHUR (v1.31.2) (30) and QIIME (v1.8.0) (28) to evaluate alpha and beta diversity, respectively. Bar and Heat maps of different taxonomic levels were drawn using R package v3.4.1 and the R package “gplots,” respectively. Linear discriminant analysis effect size (LefSe) was used to conducted LEfSe cluster or LDA analysis. In addition, the Wilcox-test or Kruskal-Test was used as statistical analysis for significant species.

### Blood Parameters Measurements

Blood samples were collected for the measurement of biomarkers of immune function and inflammation. The laser flow cytometry was used to measure white blood cell count and the respective subtypes including basophils, eosinophils, neutrophils, lymphocytes, and monocytes. Immunoturbidimetry was used to determine C-reactive protein and immunoglobulin (Ig) G and IgM. The hematology analysis was completed by Adicon Clinical Laboratories, Hangzhou, China. In our study, 0.1 g/L was used as if the value of C-reactive protein had been measured <0.2 g/L occurs (31).

### Statistical Analysis

All data analyses were performed using SPSS Statistics version 23.0 (SPSS Inc., Chicago, IL, USA). The Kolmogorov-Smirnov test was used to assess the normal distribution of variables before statistical analysis. One-way analysis of variance with normally distributed data or the Kruskal-Wallis test with non-normally distributed data was used to compare the composition of gut microbiota and blood biomarkers of immune function among elite athletes and general college students. In addition, analysis of covariance (ANCOVA) was used to evaluate whether the difference of gut microbiota and blood immune indicators among participants is independent of dietary status which included weekly intake of cereals, vegetables and fruits, meat, beans and nuts, fatty food, and alcohol. The ANCOVA is usually applied to test whether the independent variable still influences the dependent variable after the influence of the covariate has been removed (32). Pearson's correlation was used to examine the relationship between blood immune or inflammatory biomarkers and gut microbiota composition in all participants. The continuous variables were expressed as mean ± SD or SEM as appropriate, and percentages for categorical variables. A *p-*value < 0.05 was considered as an indication of a significant difference.

## Results

### Characteristics of Participants

A total of 66 young adults were enrolled in the present study. Basic information of all participants is shown in Table 1. There was no significant difference in the body mass index among the three groups. However, the Athlete group had higher muscle mass (*p* = 0.002) and lower fat mass (*p* = 0.002) than other groups. In terms of physical activity, there was a significant difference in total physical activity per week among the three groups (*p* < 0.001). As shown in Supplementary Table S1, only the HPA group had job-related physical activity, and the HPA group also participated for a longer total time in transportation and housework physical activity per week. The total time of participating in moderate and high-intensity physical activities in the Athlete group was longer than that in other groups per week (estimated to be higher than in the HPA group by 4-fold and in the LPA group by 150-fold) concerning leisure-time physical activity according to IPAQ. However, the LPA group participated for a longer total time in walking and sitting per week. With regard to physical function, the performance of both hands grip strength (*p* = 0.008 for the left hand, *p* = 0.002 for right hand), standing long jump (*p* < 0.001), sit-and-reach (*p* < 0.001), one-leg standing (right) with eyes closed (*p* = 0.007), and Harvard step test (*p* < 0.001) in the Athlete group were significantly better than the LPA group. Meanwhile, the Athlete group performed significantly better than the HPA group in the standing long jump (*p* < 0.001) and Harvard step test (*p* = 0.003). In addition, there were significant differences in the sit-and-reach (*p* = 0.019) and Harvard step test (*p* = 0.002) in the HPA group compared with the LPA group. In terms of diet, athletes tended to have higher intakes of almost all food items than the other two groups, except beans and nuts. Specifically, the intake of cereals in the Athlete (*p* < 0.001) and HPA groups (*p* = 0.017) was significantly higher than that in the LPA group. Furthermore, the intake of vegetables and fruits, as well as meat in the Athlete group was significantly higher than in the HPA group (*p* = 0.018, *p* < 0.001, respectively) and the LPA group (*p* < 0.001, *p* < 0.001, respectively).

**Table 1:** Characteristics of the participants.

**Characteristics**	**Athlete group**	**HPA group**	**LPA group**
	**(*n* = 22)**	**(*n* = 22)**	**(*n* = 22)**
Age, year (SD)	21.55 ± 2.42	21.27 ± 2.47	21.27 ± 2.51
Male (%)	12 (54.5)	12 (54.5)	12 (54.5)
BMI, kg/m^2^ (SD)	20.67 ± 5.26	22.28 ± 2.71	21.89 ± 2.81
PA, METs· hour/week	138.50 ± 108.26	59.13 ± 29.96	20.25 ± 13.17
Muscle mass, kg (SD)	27.45 ± 6.51	23.31 ± 6.44	20.52 ± 6.02
Fat mass, kg (SD)	9.70 ± 4.41	12.99 ± 4.76	15.06 ± 6.72
Fat percentage, % (SD)	14.30 ± 5.26	20.47 ± 6.67	24.08 ± 8.46
Grip strength, kg (SD)			
Left	37.34 ± 9.81	33.41 ± 10.42	29.22 ± 9.33
Right	39.15 ± 10.58	34.84 ± 11.70	29.71 ± 7.77
Standing long jump, m (SD)	2.46 ± 0.29	1.89 ± 0.32	1.77 ± 0.34
Sit-and-reach, cm (SD)	18.26 ± 8.68	14.54 ± 9.07	8.33 ± 7.82
One-leg standing with eyes closed, sec. (SD)			
Left	30.42 ± 30.07	24.67 ± 23.21	24.39 ± 26.41
Right	35.13 ± 31.24	24.72 ± 23.27	15.57 ± 10.36
Harvard step test (SD)			
Completed, *n* (%)	21 (95.45)	15 (68.18)	7 (31.82)
Harvard step index	96.56 ± 18.43	76.75 ± 18.81	56.39 ± 24.75
Daily diet, g/d (SD)			
Cereals	164.07 ± 142.82	122.70 ± 111.51	44.09 ± 29.50
Vegetables and fruits	320.38 ± 225.33	201.82 ± 128.26	120.08 ± 107.19
Meat	642.65 ± 438.48	319.17 ± 207.61	390.34 ± 339.50
Beans and nuts	31.06 ± 36.27	18.41 ± 22.43	34.55 ± 46.32
Fatty food	62.35 ± 74.98	39.47 ± 49.02	37.95 ± 45.93
Alcohol	41.36 ± 99.61	10.98 ± 29.67	24.31 ± 101.23

*PA, physical activity; HPA, high physical activity; LPA, low physical activity; BMI, body mass index; SD, Standard deviation*.

### Gut Microbial Diversity

There were a total of 9,512,790 (9.5 million) 16S rRNA reads from all sixty-three isolated fecal DNA. After quality filtering, 136,836 effective sequences were collected from each fecal sample and the effective rate is 95.80%. After removal of singletons, a total of 11,212 (Athlete = 3,853, HPA = 3,636, LPA = 3,723) operational taxonomic units (OTUs) were identified using 97% sequence similarity. The average number of observed OTUs was 170 per sample. No significant differences in the OTUs were observed among the three groups (*p* = 0.934).

Alpha diversity is the analysis of species diversity in a single microbiota sample, including observed species index, richness (Chao and Ace index), diversity (Shannon and Simpson index), and good coverage index (30). Table 2 shows that there was no statistically significant difference in alpha diversity among the Athlete, HPA, and LPA groups.

**Table 2:** Difference in alpha diversity of gut microbiota composition among athletes and young adults.

	**Athlete group**	**HPA group**	**LPA group**	
	**(*n* = 22)**	**(*n* = 22)**	**(*n* = 22)**	
**Alpha diversity**				
	**mean** **±SEM**	**mean** **±SEM**	**mean** **±SEM**	***p-value***
sobs^†^	175.14 ± 12.50	165.27 ± 8.30	169.23 ± 10.29	0.934
chao^†^	202.94 ± 13.55	197.07 ± 9.56	200.53 ± 13.24	0.925
Ace^†^	207.91 ± 13.63	196.60 ± 9.16	194.87 ± 12.59	0.712
Shannon^†^	2.52 ± 0.16	2.60 ± 0.15	2.64 ± 0.14	0.924
Simpson^†^	0.20 ± 0.03	0.19 ± 0.03	0.19 ± 0.03	0.936
Coverage^‡^	1.00 ± 0.00	1.00 ± 0.00	1.00 ± 0.00	0.744

*HPA, high physical activity; LPA, low physical activity. SEM, Standard error of the mean*.

† *Kruskal–Wallis test*;

‡ *One-way analysis of variance*.

Regarding beta diversity, no significant differences were observed in the Principal Coordinates Analysis (PCoA) based on unweighted-Unifrac distance metrics (*p* = 0.845) or weighted-Unifrac distance metrics (*p* = 0.372) (Supplementary Figure S1). However, the beta weighted-Unifrac heatmap showed a trend of the cluster among the three groups (Supplementary Figure S2). The taxonomy of the three groups of gut microbiomes is different. Linear discriminant analysis effect size (LEfSe) showed that the Athlete group enriched nine taxa, the HPA group enriched one taxon, and the LPA group enriched two taxa (*p* < 0.05). Specifically, the most abundant microbiota of the Athlete and LPA group was the *Firmicutes*, while the *Proteobacteria* in the HPA group (Supplementary Figure S3). These bacterial taxa indicated significant enrichment due to the linear discriminant analysis (LDA) score >2 or <-2 (33).

### Gut Microbiota Composition at Phylum and Class Levels

**Figure 2:**
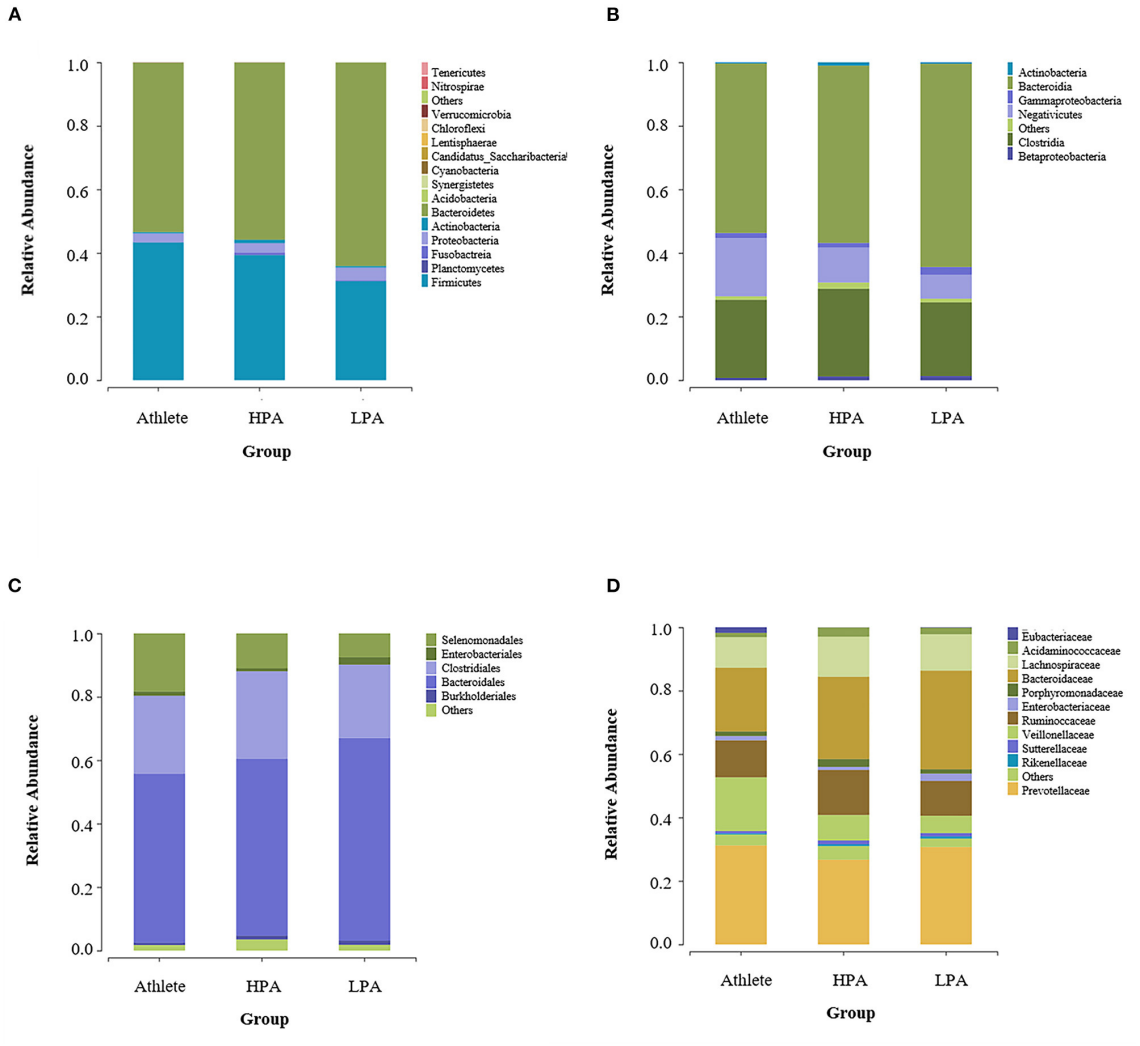
Differences of gut microbiota relative abundance at the phylum, class, order, and family levels among three groups. Relative abundance represents the proportion of each species in each group. **(A)** phylum level; **(B)** class level; **(C)** order level; and **(D)** family level. Athlete, Athlete group; HPA, High physical activity group; LPA, Low physical activity group.

The gut microbiota composition at the phylum level among three groups was represented in Figure 2A. In total, 16 phyla were observed in the intestines of different groups. Of these, 12 phyla were observed in Athlete and HPA groups, while 14 phyla were observed in the LPA group. The abundant phyla were the *Bacteroidetes* (52.53 in the Athlete group, 55.35 in the HPA group, and 62.81% in the LPA group) and the *Firmicutes* (43.99 in the Athlete group, 39.67 in the HPA group, and 32.14% in the LPA group) among three groups. However, there were no significant differences in the abundance of the *Bacteroidetes* and the *Firmicutes* among the three groups (*p* = 0.278, *p* = 0.164, respectively, data not shown). Similarly, the relative abundance of *Firmicutes*: *Bacteroidetes* ratio was evaluated, and no difference was observed among the three groups (*p* = 0.417, data not shown).

Figure 2B shows the gut microbiota composition at the class level among the three groups. *Bacteroidia* (52.63 in the Athlete group, 55.34 in the HPA group, and 62.71% in the LPA group), *Clostridia* (25.68 in the Athlete group, 28.46 in the HPA group, and 24.03% in the LPA group), and *Negativicutes* (17.84 in the Athlete group, 10.39 in the HPA group, and 7.62% in the LPA group) were the dominant genus among the three groups.

### Gut Microbiota Composition at Order and Family Levels

At the order level, *Bacteroidales* (52.53 in the Athlete group, 55.34 in the HPA group, and 62.71% in the LPA group), *Clostridiales* (25.76 in the Athlete group, 28.46 in the HPA group, and 24.03% in the LPA group), *Selenomonadales* (17.84 in the Athlete group, 10.39 in the HPA group, and 7.62% in the LPA group) were the dominant genus among the three groups (Figure 2C). For family level, *Prevotellaceae* (29.60 in the Athlete group, 25.32 in the HPA group, and 28.61% in the LPA group), *Bacteroidaceae* (20.88 in the Athlete group, 26.81 in the HPA group, and 31.92% in the LPA group), *Ruminococcaceae* (12.28 in the Athlete group, 14.67 in the HPA group, and 11.56% in the LPA group) were the dominant genus among the three groups (Figure 2D).

### Gut Microbiota Composition at Genus and Species Levels

**Figure 3:**
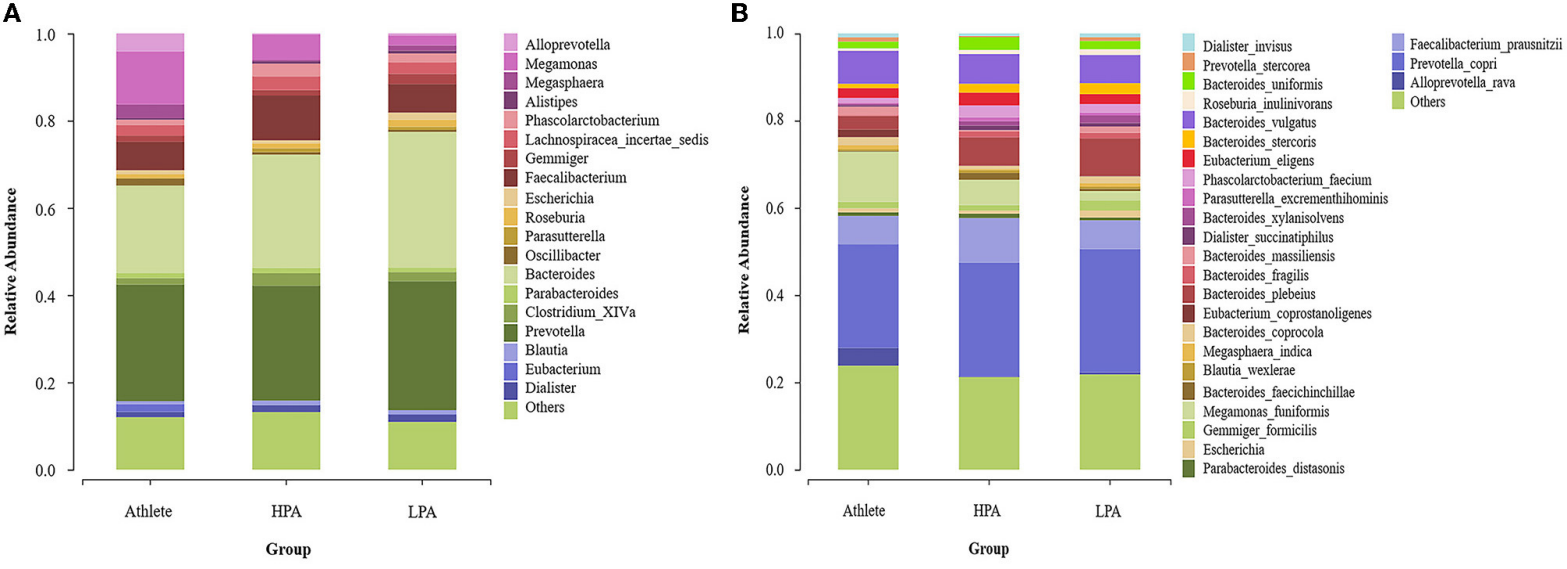
Differences of gut microbiota relative abundance at the genus and species level among three groups. Relative abundance represents the proportion of each species in each group; **(A)** genus level; **(B)** species level; Athlete, Athlete group; HPA,High physical activity group; LPA, Low physical activity group.

Figure 3A indicated the gut microbiota composition at the genus level among the three groups. *Prevotella* (20.88 in Athlete group, 26.81 in the HPA group, and 31.92% in the LPA group), *Bacteroides* (24.96 in Athlete group, 25.01 in the HPA group, and 27.41% in the LPA group), *Faecalibacterium* (6.86 in Athlete group, 10.57 in the HPA group and 6.88% in LPA group) and *Megamonas* (11.67 in the Athlete group, 5.15 in the HPA group and 2.24% in the LPA group) were the dominant genus among three groups.

At the species level, *Prevotella_copri* (21.80 in the Athlete group, 24.64 in the HPA group, and 26.11% in the LPA group), *Faecalibacterium_prausnitzii* (6.86 in the Athlete group, 10.57 in the HPA group, and 6.88% in the LPA group), *Bacteroides_vulgatus* (7.74 in Athlete group, 6.78 in HPA group, and 6.73% in LPA group), *Bacteroides_plebeius (*3.32% in the Athlete group, 6.87 in the HPA group, and 8.61% in the LPA group), and *Megamonas_funiformis* (11.07 in the Athlete group, 5.09 in the HPA group, and 2.11% in the LPA group) were the dominant genus among three groups (Figure 3B).

### Differences in Gut Microbiota Composition Among Athletes and Young Adults With Different Physical Activity Levels

A total of 25 taxa of gut microbiota were observed significant differences among the groups in the non-adjusted model. In the Athlete group, the abundance of *Lentisphaerae (Victivallaceae* belongs to *Lentisphaerae*; *p* = 0.045), *Clostridiaceae* (*p* = 0.029), *Megamonas* (*p* = 0.020), *Romboutsia* (*p* = 0.038), *Campylobacter_jejuni* (*p* = 0.045), and *Paraprevotella_xylaniphila* (*p* = 0.045), were higher. In the LPA group, the abundance of *Erysipelotrichia* (*p* = 0.040), *Bilophila* (*p* = 0.034), and *Faecalicoccus* (*p* = 0.008) were higher. In addition, the abundance of *Parasutterella* (*p* = 0.026), *Arcobacter_halophilus* (*p* = 0.045), *Parasutterella_excrementihominis* (*p* = 0.023) and *Clostridium_spiroforme* (*p* = 0.017) were higher in the HPA group.

Moreover, we re-analyzed the bacteria with differences between groups after dietary adjustment, and the results show that there were no group differences in most bacteria after adjusted diet intake. Only the abundance of *Clostridiaceae* (*p* = 0.029), *Bilophila* (*p* = 0.011), *Faecalicoccus* (*p* = 0.050), and *Bilophila_wadsworthia* (*p* = 0.011) were still different between groups. In addition, although no differences were observed between the three groups, two bacteria (*Campylobacter_jejuni* and *Megamonas_rupellensis*) showed differences among participants. *Campylobacter_jejuni* was higher in the Athlete group than in the LPA group (*p* = 0.050), and *Megamonas_rupellensis* sp. was higher in the Athlete group than in the HPA group (*p* = 0.029) (Table 3).

**Table 3:** Phylotypes with significant difference among athletes and young adults before and after adjusting dietary status.

		**Non-adjusted model^†^**				**Dietary status adjusted model^‡^**			
**Taxa**	**specific**	**Athlete group**	**HPA group**	**LPA group**		**Athlete group**	**HPA group**	**LPA group**	
		**(*n* = 22)**	**(*n* = 22)**	**(*n* = 22)**		**(*n* = 22)**	**(*n* = 22)**	**(*n* =22)**	
		**Mean ±SEM**	**Mean ±SEM**	**Mean ±SEM**	***p-value***	**Mean ±SEM**	**Mean ±SEM**	**Mean ±SEM**	***p-value***
Phylum	Lentisphaerae	0.004 ± 0.004	0.000 ± 0.000	0.000 ± 0.000	0.045	0.005 ± 0.002	0.000 ± 0.002	−0.001 ± 0.002	0.242
Class	Erysipelotrichia	0.143 ± 0.036	0.110 ± 0.028	0.221 ± 0.051^#^	0.040	0.129 ± 0.045	0.115 ± 0.042	0.231 ± 0.044	0.148
	Lentisphaeria	0.004 ± 0.004	0.000 ± 0.000	0.000 ± 0.000	0.045	0.005 ± 0.002	0.000 ± 0.002	−0.001 ± 0.002	0.242
Order	Erysipelotrichales	0.143 ± 0.036	0.110 ± 0.028	0.221 ± 0.051^#^	0.040	0.129 ± 0.045	0.115 ± 0.042	0.231 ± 0.044	0.148
	Victivallales	0.004 ± 0.004	0.000 ± 0.000	0.000 ± 0.000	0.045	0.005 ± 0.002	0.000 ± 0.002	−0.001 ± 0.002	0.242
Family	Clostridiaceae	0.144 ± 0.070	0.023 ± 0.008^*^	0.026 ± 0.014	0.029	0.180 ± 0.049	0.014 ± 0.046^*^	−0.002 ± 0.048^*^	0.029
	Erysipelotrichaceae	0.143 ± 0.036	0.110 ± 0.028	0.221 ± 0.051^#^	0.040	0.129 ± 0.045	0.115 ± 0.042	0.231 ± 0.044	0.148
	Victivallaceae	0.004 ± 0.004	0.000 ± 0.000	0.000 ± 0.000	0.045	0.005 ± 0.002	0.000 ± 0.002	−0.001 ± 0.002	0.242
Genus	Bilophila	0.100 ± 0.032	0.101 ± 0.029	0.249 ± 0.053	0.034	0.113 ± 0.044	0.076 ± 0.041	0.261 ± 0.043^*##^	0.011
	Faecalicoccus	0.037 ± 0.024	0.005 ± 0.002	0.092 ± 0.038^##^	0.008	0.022 ± 0.028	0.010 ± 0.026	0.103 ± 0.028^*^	0.050
	Megamonas	11.670 ± 3.898	5.154 ± 4.276^*^	2.239 ± 0.776	0.020	11.254 ± 3.871	6.970 ± 3.622	0.839 ± 3.794	0.206
	Parasutterella	0.257 ± 0.144	0.969 ± 0.276^*^	0.703 ± 0.344	0.026	0.360 ± 0.316	1.029 ± 0.295	0.539 ± 0.309	0.290
	Romboutsia	0.134 ± 0.034	0.062 ± 0.017^*^	0.111 ± 0.056	0.038	0.127 ± 0.046	0.071 ± 0.043	0.109 ± 0.045	0.661
	Victivallis	0.004 ± 0.004	0.000 ± 0.000	0.000 ± 0.000	0.045	0.005 ± 0.002	0.000 ± 0.002	−0.001 ± 0.002	0.242
Species	Arcobacter_halophilus	0.000 ± 0.000	0.001 ± 0.001	0.000 ± 0.000	0.045	0.000 ± 0.000	0.001 ± 0.000	0.000 ± 0.000	0.350
	Bilophila_wadsworthia	0.100 ± 0.032	0.101 ± 0.029	0.249 ± 0.053	0.034	0.113 ± 0.044	0.076 ± 0.041	0.261 ± 0.043^*##^	0.011
	Campylobacter_jejuni	0.046 ± 0.045	0.000 ± 0.000	0.000 ± 0.000	0.045	0.072 ± 0.030	−0.007 ± 0.028	−0.020 ± 0.030^*^	0.103
	Clostridium_spiroforme	0.000 ± 0.000	0.002 ± 0.001^*^	0.000 ± 0.000	0.017	0.000 ± 0.001	0.002 ± 0.001	0.000 ± 0.001	0.139
	Megamonas_funiformis	11.065 ± 3.721	5.092 ± 4.240^*^	2.109 ± 0.733	0.019	10.661 ± 3.773	6.879 ± 3.530	0.726 ± 3.698	0.216
	Megamonas_rupellensis	0.125 ± 0.046	0.004 ± 0.003^*^	0.041 ± 0.032	0.036	0.125 ± 0.038	0.005 ± 0.035^*^	0.039 ± 0.037	0.087
	Paraprevotella_xylaniphila	0.100 ± 0.062	0.000 ± 0.000	0.000 ± 0.000	0.045	0.038 ± 0.035	−0.015 ± 0.033	0.077 ± 0.034	0.167
	Parasutterella_excrementihominis	0.253 ± 0.144	0.969 ± 0.276^*^	0.699 ± 0.344	0.023	0.357 ± 0.316	1.030 ± 0.295	0.533 ± 0.309	0.285
	Romboutsia_sedimentorum	0.134 ± 0.034	0.062 ± 0.017^*^	0.111 ± 0.056	0.038	0.127 ± 0.046	0.071 ± 0.043	0.109 ± 0.045	0.661
	Rothia_aeria	0.000 ± 0.000	0.004 ± 0.000	0.000 ± 0.000	0.045	0.000 ± 0.000	0.000 ± 0.000	0.000 ± 0.000	0.166
	Victivallis_vadensis	0.004 ± 0.003	0.000 ± 0.000	0.000 ± 0.000	0.045	0.005 ± 0.002	0.000 ± 0.002	−0.001 ± 0.002	0.242

*HPA, high physical activity; LPA, low physical activity; SEM, Standard error of the mean*.

* *p < 0.05 when HPA or LPA group compared with Athlete group*.

# *p < 0.05 when LPA group compared with HPA group*;

## *p < 0.01 when LPA group compared with HPA group*.

† *Kruskal–Wallis test*;

‡ *Analysis of covariance*.

### Association of Serum Immune Function Biomarkers With Gut Microbiota

Concerning immune function biomarkers, a significantly higher absolute neutrophil count (*p* = 0.004) and white blood cell count (*p* = 0.014) were observed in the LPA group than in other groups in the non-adjusted model. In addition, the HPA group had a higher absolute basophil count (*p* = 0.022). When adjusting for dietary status, almost all these significant differences disappeared and only a significantly higher absolute count of basophils was observed in the HPA group than in the athlete group (*p* = 0.037) (Table 4).

**Table 4:** Blood biomarkers with significant difference among athletes and young adults before and after adjusting dietary status.

	**Non-adjusted model**				**Dietary status adjusted model^Ψ^**			
**Blood parameters**	**Athlete group**	**HPA group**	**LPA group**		**Athlete group**	**HPA group**	**LPA group**	
	**(*n* = 22)**	**(*n* = 22)**	**(*n* = 22)**		**(*n* = 22)**	**(*n* = 22)**	**(*n* =22)**	
	**Mean ±SEM**	**Mean ±SEM**	**Mean ±SEM**	***p-value***	**Mean ±SEM**	**Mean ±SEM**	**Mean ±SEM**	***p-value***
White body cell, (10^9^/L)^†^	4.96 ± 0.20	5.53 ± 0.23	6.45 ± 0.41^*^	0.014	5.18 ± 0.34	5.57 ± 0.32	6.19 ± 0.33	0.148
Basophils, (10^9^/L)^†^	0.027 ± 0.002	0.039 ± 0.003^*^	0.034 ± 0.003	0.022	0.028 ± 0.003	0.038 ± 0.003^*^	0.034 ± 0.003	0.111
Eosinophils, (10^9^/L)^†^	0.136 ± 0.020	0.136 ± 0.015	0.142 ± 0.019	0.886	0.130 ± 0.022	0.141 ± 0.020	0.144 ± 0.021	0.902
Neutrophils, (10^9^/L)^‡^	2.30 ± 0.15	2.93 ± 0.18^*^	3.32 ± 0.27^*^	0.004	2.46 ± 0.23	2.94 ± 0.22	3.15 ± 0.23	0.147
Lymphocytes, (10^9^/L)^†^	2.19 ± 0.10	2.10 ± 0.10	2.62 ± 0.17	0.062	2.25 ± 0.15	2.13 ± 0.14	2.54 ± 0.15	0.151
Monocytes, (10^9^/L)^‡^	0.303 ± 0.016	0.326 ± 0.022	0.343 ± 0.023	0.392	0.311 ± 0.024	0.330 ± 0.022	0.331 ± 0.023	0.818
Immunoglobulin G, (g/L)^‡^	11.29 ± 0.26	12.07 ± 0.37	11.97 ± 0.47	0.285	11.18 ± 0.44	12.14 ± 0.41	12.00 ± 0.43	0.279
Immunoglobulin M, (g/L)^‡^	1.46 ± 0.09	1.34 ± 0.09	1.52 ± 0.12	0.412	1.46 ± 0.12	1.34 ± 0.11	1.53 ± 0.12	0.490
C-reactive protein, (g/L)^†^	0.81 ± 0.22	1.14 ± 0.53	0.74 ± 0.10	0.122	0.97 ± 0.39	1.27 ± 0.37	0.45 ± 0.39	0.324

*HPA, high physical activity; LPA, low physical activity; SEM, Standard error of the mean*.

* *p < 0.05 when HPA or LPA group compared with Athlete group*.

† *Kruskal–Wallis test in non-adjusted model*;

‡ *One-way analysis of variance in non-adjusted model*;

Ψ *Analysis of covariance in dietary status adjusted model*.

**Figure 4:**
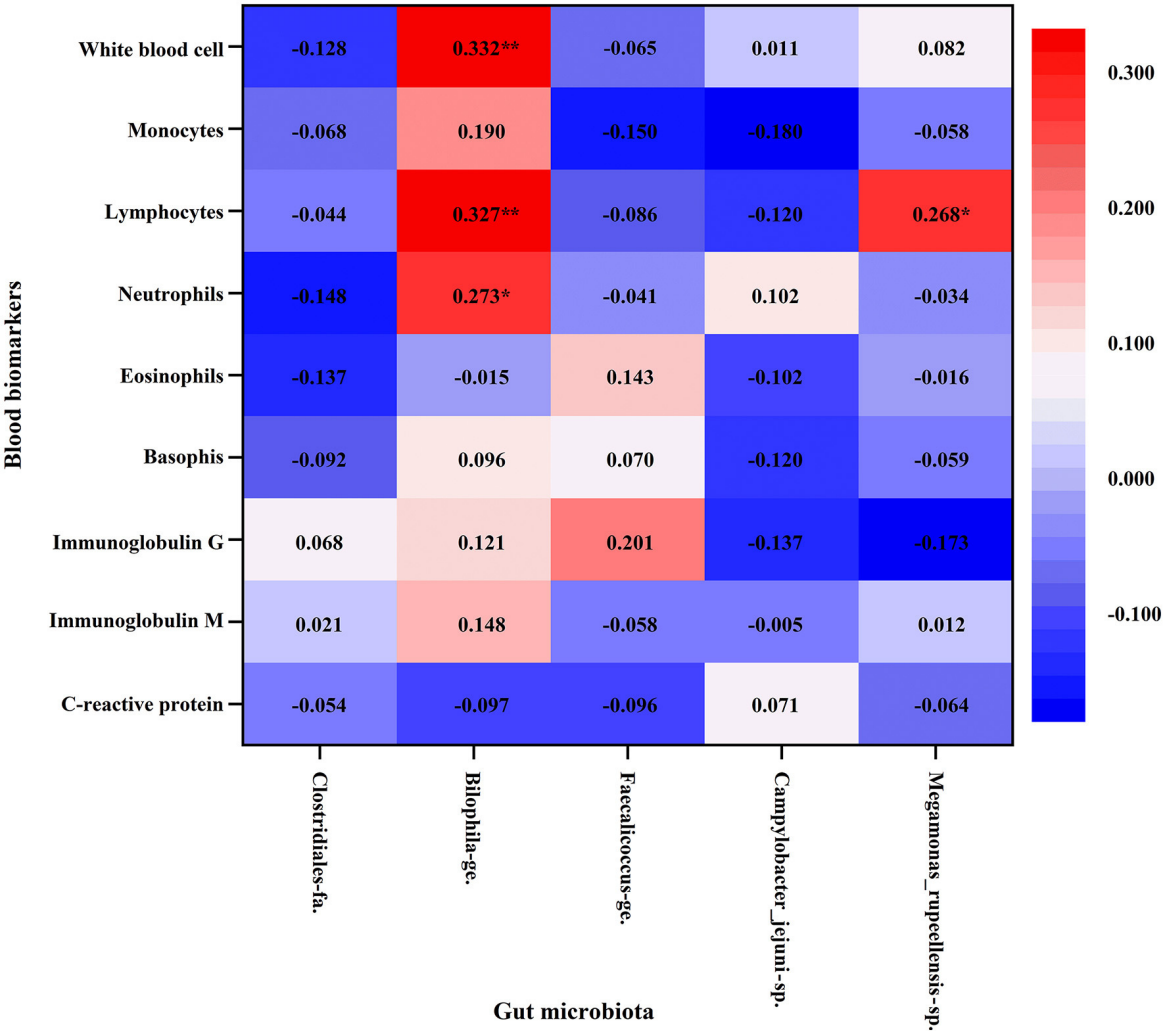
Association between blood biomarkers of immune function and gut microbiota with Pearson's correlation analysis. Athlete, Athlete group; HPA, High physical activity group; LPA, Low physical activity group. *p < 0.05; **p < 0.01.

Pearson's correlation was conducted to further explore the relationship between gut microbiota, inflammation, and immunity (Figure 4). It was shown that *Bilophila* genus (*Bilophila_wadsworthia*) had positive linear correlation with the absolute neutrophil (*r* = 0.273, *p*=0.027), lymphocyte count (*r* = 0.327, *p* = 0.007), and white blood cell count (*r* = 0.332, *p* = 0.006). At the species level, *Megamonas_rupellensis* was also positively correlated with the absolute lymphocyte count (*r* = 0.268, *p* = 0.030).

## Discussion

This matching study investigated the characteristics of gut microbiota at all levels (including phylum, class, order, family, genus, and species) among elite athletes and general young adults with different physical activity levels. Beta diversity tended to cluster between groups with different physical activity levels, while no significant difference was observed in both alpha and beta diversity. Meanwhile, a higher abundance of several bacteria was observed in elite athletes or physically inactive young adults. Moreover, it is inferred that dietary status may be a key factor to be considered when analyzing the association between physical activity and gut microbiota.

In this study, the elite athletes had a significantly higher level of total physical activity and moderate-to-vigorous intensity physical activity, as well as lower sitting time compared to general young adults. It is worth noting that these gut microbiological differences between people with different levels of physical activity have also been observed in previous studies. Specifically, the *Bacteroides* and *Prevotella* were more abundant in top Polish endurance athletes compared to sedentary participants (34). The elite athletes and youth non-elite athletes have different taxonomical functional and phenotypic compositions of the gut microbial community. *Clostridiales* and *Faecalibacterium* were particularly enriched in the elite athletes (35). Senior orienteers were found to have a more homogeneous microbiota and more abundant *Faecalibacterium prausnitzii* than the community-dwelling older adults (36). Indeed, our results indicate partial disparity of gut microbiota composition among elite athletes and young adults with different physical activity, particularly after adjusting dietary status.

Diet is an important factor affecting gut microbiota (17). Some previous studies confirmed that the different dietary ingredients have a substantial impact on the gut microbiota (37). Specifically, high-fiber feeding can reshape gut microbiota and promote the release of short-chain fatty acids (SCFAs), which play a role in maintaining the normal functions of the innate and adaptive immune system (38, 39). The intervention of low-calorie weight loss on a high-fat diet induced the growth of bile-resistant bacteria and reduction of bacteria associated with inflammation in humans (40). Moreover, in athletes, high-protein diets may be negatively associated with the diversity of gut microbiota, and a decreased relative abundance of short-chain fatty acid-producing commensal bacteria was observed in a high protein low carbohydrates diet for athletes in resistance sports (41). It is worth noting that athletes often consume a diet that differs from the general population, including in dietary diversity and total energy consumption. The eating habits of athletes are even different between different events (e.g., increased protein intake in resistance-trained athletes or carbohydrate intake in endurance athletes) (42). Previous studies have shown that there is a rapid change in the composition of the gut microbial and increased abundance of *Alistipes, Bilophila*, and *Bacteroides* after consuming a high-fat/protein diet for 5 days (43). Thus, there is a need to consider the influence of an athletic diet when comparing the differences in gut microbiota among athletes of different sports, as well as between athletes and the general population. In our study, 19 taxa of gut microbiota showed no statistical differences, the remaining 6 taxa (*Clostridiaceae, Bilophila, Faecalicoccus, Bilophila_wadsworthia, Campylobacter_jejuni, Megamonas_rupellensis*) observed significant differences among the groups after adjusting the covariates of dietary intake. These changes show that dietary intake is also a particularly important factor in regulating microbiota composition of the gut between elite athletes and general young adults.

Previous studies indicated that some abundant gut microbiota in physically inactive young adults of this study may be closely associated with inflammation and immunity. For example, a high abundance of *Bilophila*_*wadsworthia* (belong to *Bilophila* genus) caused a systemic inflammatory response in mice that included elevated IL-6 (44), which was enriched in patients with Behcet's disease (45) and can promote a Th1-mediated immune response in dietary-fat-induced colitis (46). In addition, a recent study demonstrated that *Faecalicoccus* was considered to be an important factor in the classification of subjects in Crohn's disease and enriched in patients with Crohn's disease (47). Our study showed that a higher abundance of *Bilophila* and *Faecalicoccus* was observed in young adults with insufficient physical activity. It is worth noting that *Bilophila* genus was positively associated with the counts of circulating white blood cells and its subtypes such as neutrophils and lymphocytes. The bacterium *Bilophila_wadsworthia* can erode the mucus layer on colon surfaces and allow more bacterial flora to approach lining cells, then resulting in inflammation (48). These findings suggest that physical inactivity may be correlated to pro-inflammatory gut bacteria.

It is well-known that physical inactivity is associated with high-grade systemic inflammation (49) and low immune function (50). However, the mechanism by which physical inactivity induces a reduction of immune function has not been fully elucidated. One possible underlying mechanism is that physical inactivity or low physical activity may lead to impaired ability to store fat and inflammation of subcutaneous adipose tissue (51, 52). In addition, the subjects with low physical activity have a higher susceptibility to infection compared to regular moderate exercise subjects (53). The reason may be that the growth hormone, cortisol, adrenaline, prolactin, and other factors showed a decreased release, leading to attenuated immunomodulatory effects in the subjects with physical inactivity (54).

It is well known that, the white blood cell, comprising neutrophils, eosinophils, basophils, lymphocytes, and monocytes, play an important role in the implementation of immune function and anti-infection in humans (55). The leukocyte count can increase in response to infections. Evidence indicated that patients with severe COVID-19 infection tend to have a higher leukocyte count in the Chinese population (56). Moreover, the elevated leukocyte count, particularly neutrophil and monocyte count were reliable inflammatory markers within the normal range, even in physically healthy individuals (57–59). Thus, it may reduce the body's inflammation and the risk of inflammation-induced diseases if the number of leukocytes can be kept at an appropriate level. Studies have reported regular exercise, particularly in more moderate-to-vigorous physical activity, can have an anti-inflammatory effect and reduce the odds of elevated white blood cell count (51, 60, 61). Although previous studies focused on elite athletes who practice water sports (62), cyclists who participate in intense endurance exercise (63) have higher levels of inflammation and low immune function. The reason for these findings could be that exercise-induced immunosuppression makes elite athletes more susceptible to infection symptoms after short-term acute exercise. Our results showed that athletes participating in regular exercise training have better immune function reflected by lower leukocyte count. Conversely, the *Clostridiaceae*, a bacterium associated with inflammation (64) was more abundant in athletes. As another bacterium relatively abundant in athletes, *Megamonas_rupellensis* also showed a positive correlation with circulating lymphocytes. These results may be interpreted as injury-induced local inflammation when the athletes are susceptible to sports injury. Evidence showed that muscle injury in basketball players can lead to increased levels of inflammation (65). Due to the lack of data on acute or chronic sports injury among athletes, these speculations could not be confirmed in this study and need to be further clarified in subsequent work.

We have to admit that there are some limitations to this study. First, our elite athletes only include track and field athletes, and the lack of participants from other sports may affect the results of the gut microbiota in different groups, hence similar findings may not necessarily be observed. Second, there was no data on the nutrient supplementation of participants, particularly in elite athletes, hence we cannot rule out the effect of these factors on main observation results (66). Third, we did not analyze the metabolites of gut microbes, which might normally be directly involved in the human metabolism process (67), hence the in-depth interpretation of results was limited.

## Conclusion

These findings indicated that several gut bacteria are inversely associated with immune function and tended to be abundant in physically inactive people, while elite athletes are more likely to have a better immune function and specific gut bacteria composition than general young adults. It is important to note that dietary intakes are potential confounders that may affect the observed association of physical activity with gut microbiota and immune function. Further studies are warranted to determine the correlation between physical activity and gut microbiota. Moreover, considering diet, the effects and mechanisms of long-term habits with moderate and vigorous-intensity physical activity on immune indices and gut microbiota need to be further clarified.

## Data Availability Statement

The datasets presented in this article are not readily available without the consent and permission of the study participants. Requests to access the datasets should be directed to Fei Zhong, zf358235743@zju.edu.cn.

## Ethics Statement

The studies involving human participants were reviewed and approved by the Ethics Committee of Department of Psychology and Behavioral Sciences, Zhejiang University. The patients/participants provided their written informed consent to participate in this study.

## Author Contributions

YX, XZ, CW, and CH: designing the experiment. YX, FZ, XZ, and H-YL: acquisition of subjects and data. YX, FZ, XZ, H-YL, CW, and CH: statistical analyses and interpretation of data and revision of the manuscript. YX, FZ, XZ, and CH: drafting the manuscript. CH: study supervision. All authors have revised and approved the final version.

## Funding

This study was supported by the fundamental research funds (204201^*^172220192) for the Central Universities, China, and the Hundred Talents Program funding (188020^*^194221802/004/001) from Zhejiang University, China.

## Conflict of Interest

The authors declare that the research was conducted in the absence of any commercial or financial relationships that could be construed as a potential conflict of interest.

## Publisher's Note

All claims expressed in this article are solely those of the authors and do not necessarily represent those of their affiliated organizations, or those of the publisher, the editors and the reviewers. Any product that may be evaluated in this article, or claim that may be made by its manufacturer, is not guaranteed or endorsed by the publisher.

## Supplementary Material

Supplementary Figure S1 Principal Coordinate Analysis (PCoA) of gut microbiota. **(A)** unweighted-Unifrac distance metrics; **(B)** weighted-Unifrac distance metrics. Each point represents a sample, athlete group in blue, high physical activity group in orange, and low physical activity group in green. The two coordinates are plotted and further indicated the percentage of variability on the axis.

Supplementary Figure S2 Beta weighted-Unifrac heatmap. The Gradient color in the heatmap represents the degree of difference of each sample among athletes, high physical activity group, and low physical activity group. The distance between samples indicated increases gradually with the color from blue to red.

Supplementary Figure S3 Differential taxon features were analyzed by linear discriminant analysis (LDA) effect size (LEfSe) analysis among three groups. **(A)** showing differentially abundant bacterial taxa. **(B)** indicating LDA scores. Bacterial taxa indicated significantly high when LDA score >2 or LDA score <-2. Athlete, Athlete group; HPA, High physical activity group; LPA, Low physical activity group.
