# Supplementary figures and images for: Disparity of Gut Microbiota Composition Among Elite Athletes and Young Adults With Different Physical Activity Independent of Dietary Status: A Matching Study
Source: Front Nutr. 2022 Mar 18;9:843076. doi: 10.3389/fnut.2022.843076 (PMC8975590; doi:10.3389/fnut.2022.843076)

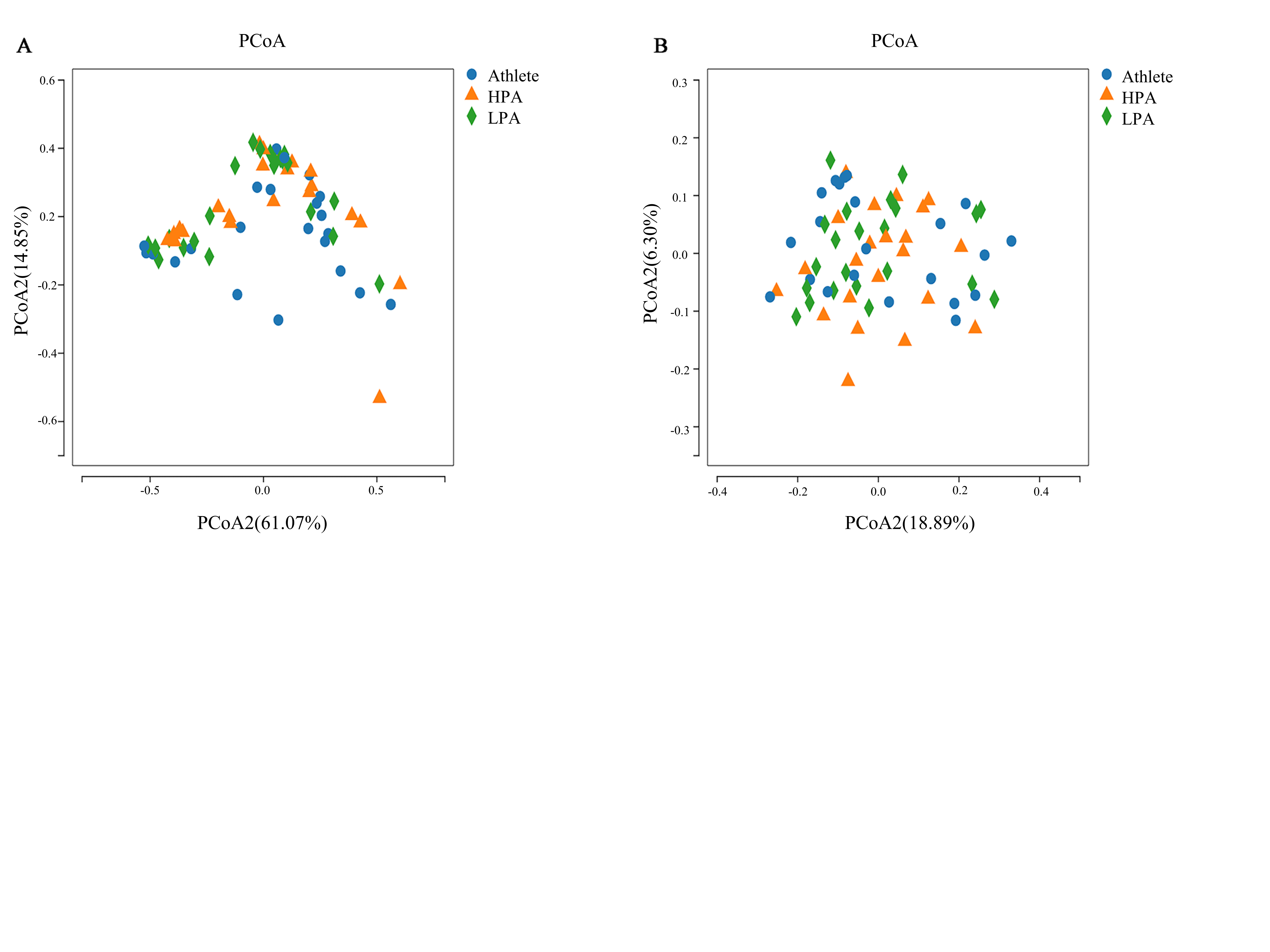

Supplement: Supplementary Figure S1 — Principal Coordinate Analysis (PCoA) of gut microbiota. (A) unweighted-Unifrac distance metrics; (B) weighted-Unifrac distance metrics. Each point represents a sample, athlete group in blue, high physical activity group in orange, and low physical activity group in green. The two coordinates are plotted and further indicated the percentage of variability on the axis. [file Image_1.tif]

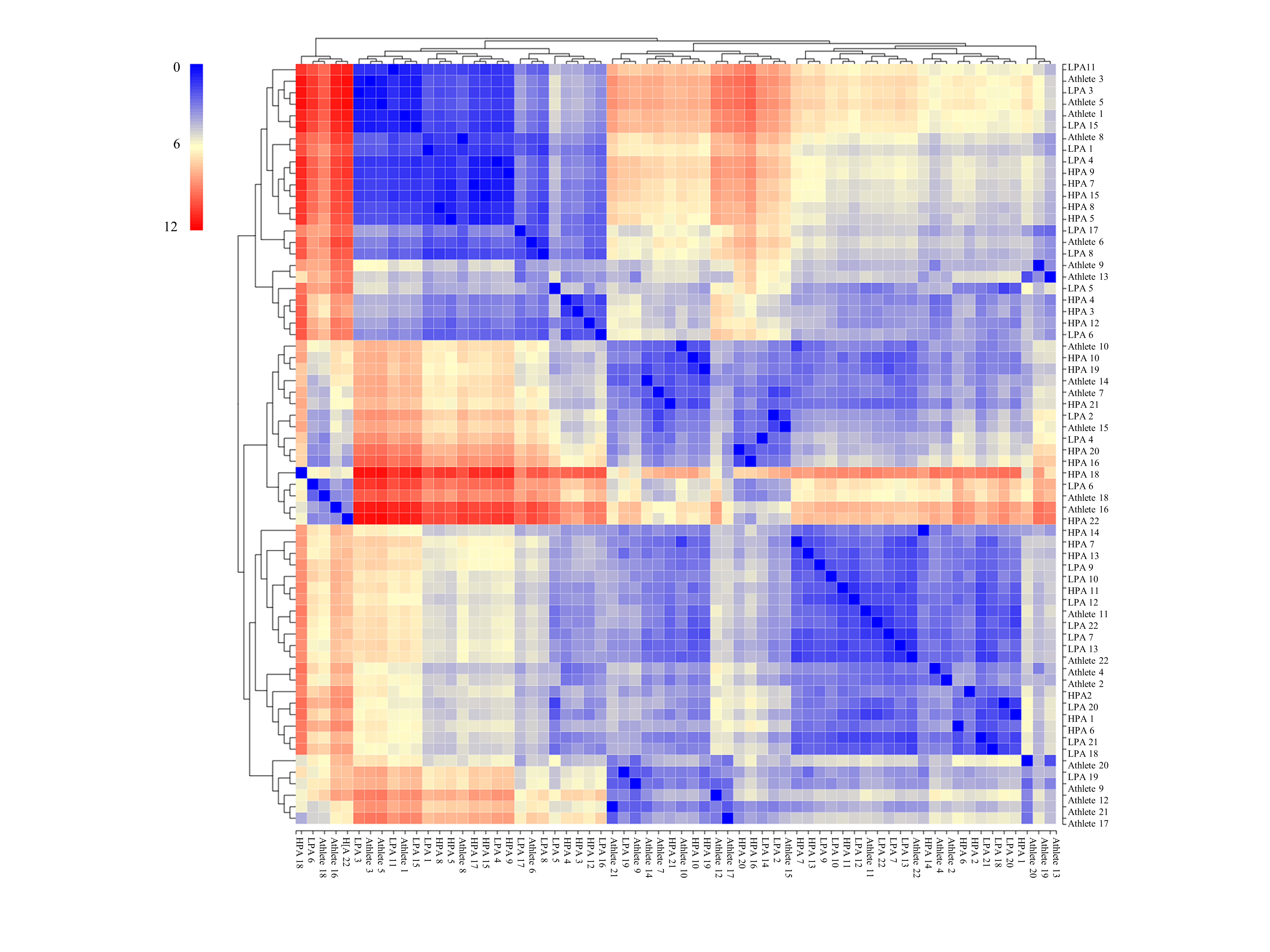

Supplement: Supplementary Figure S2 — Beta weighted-Unifrac heatmap. The Gradient color in the heatmap represents the degree of difference of each sample among athletes, high physical activity group, and low physical activity group. The distance between samples indicated increases gradually with the color from blue to red. [file Image_2.tif]

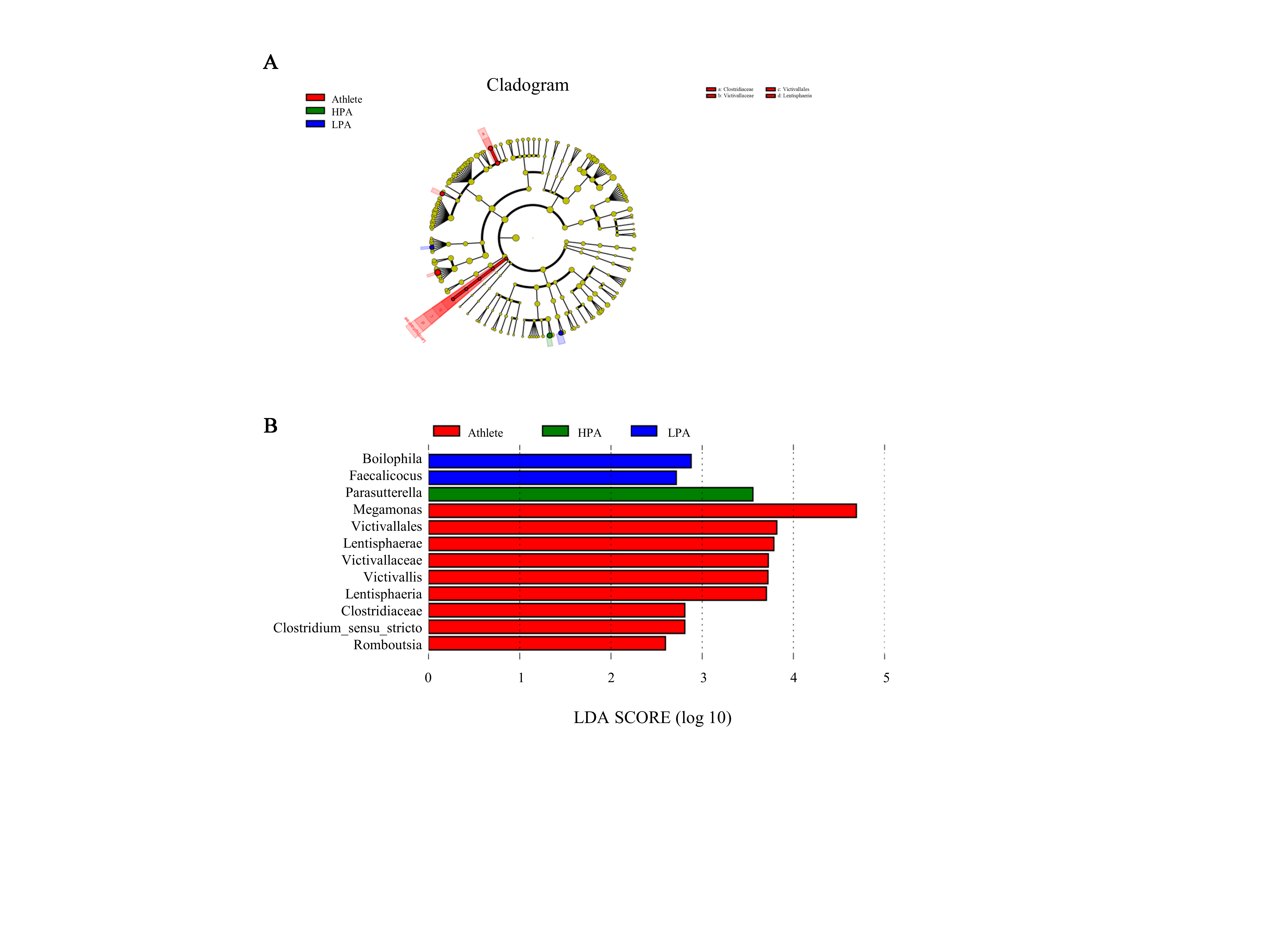

Supplement: Supplementary Figure S3 — Differential taxon features were analyzed by linear discriminant analysis (LDA) effect size (LEfSe) analysis among three groups. (A) showing differentially abundant bacterial taxa. (B) indicating LDA scores. Bacterial taxa indicated significantly high when LDA score >2 or LDA score <-2. Athlete, Athlete group; HPA, High physical activity group; LPA, Low physical activity group. [file Image_3.tif]
