# Supplementary material for: Disparity of Gut Microbiota Composition Among Elite Athletes and Young Adults With Different Physical Activity Independent of Dietary Status: A Matching Study
Source: Front Nutr. 2022 Mar 18;9:843076. doi: 10.3389/fnut.2022.843076 (PMC8975590; doi:10.3389/fnut.2022.843076)
Supplement: Supplementary file 4 [file Table_1.docx]

**Table S1** Information on physical activity elements among participants

| Characteristics | Athlete group (n=22) | HPA group (n=22) | LPA group (n=22) |
| --- | --- | --- | --- |
| Job-related PA, min/week (SD) | | | |
| Vigorous PA | 0.00±0.00 | 5.45±25.58 | 0.00±0.00 |
| Moderate PA | 0.00±0.00 | 0.00±0.00 | 0.00±0.00 |
| Walking | 0.00±0.00 | 19.09±89.54 | 0.00±0.00 |
| Transportation PA, min/week (SD) | | | |
| Cycling | 5.45±25.58 | 155.00±201.91 | 101.14±153.14 |
| Walking | 20.91±61.79 | 122.05±152.84 | 89.59±105.96 |
| Housework PA, min/week (SD) | | | |
| Vigorous PA | 0.91±4.26 | 21.36±57.92 | 0.00±0.00 |
| Moderate PA | 53.18±120.02 | 62.27±141.28 | 8.86±19.14 |
| Leisure-time PA, min/week (SD) | | | |
| Vigorous PA | 915.45±793.93 | 190.45±184.63 | 3.86±12.53 |
| Moderate PA | 143.64±269.15 | 59.55±103.81 | 3.64±17.06 |
| Walking | 44.09±84.50 | 49.77±85.59 | 80.91±117.06 |
| Sitting time, min/week (SD) | 1107.27±686.30 | 2930.36±775.28 | 3216.55±1059.79 |

Note: PA, physical activity; HPA, high physical activity; LPA, low physical activity; SD, Standard deviation.
